# Psychosocial factors related to intensive care work and the health of the nursing staff

**DOI:** 10.47626/1679-4435-2025-1406

**Published:** 2025-08-25

**Authors:** Marcelia Cristina de Oliveira, Thaís Alencar Linhares Peixoto, Maria Victória Borges Souza Lima, Renata Flavia Abreu da Silva, Amanda Guedes dos Reis, Cristiane Helena Gallasch

**Affiliations:** 1 Programa de Pós-Graduação em Enfermagem, Universidade do Estado do Rio de Janeiro (UERJ), Rio de Janeiro, RJ, Brazil; 2 Graduação em Enfermagem pela Universidade do Estado do Rio de Janeiro, Rio de Janeiro, RJ, Brazil; 3 Escola de Enfermagem Alfredo Pinto, Universidade Federal do Estado do Rio de Janeiro, Rio de Janeiro, RJ, Brazil; 4 Faculdade de Enfermagem da Universidade do Estado do Rio de Janeiro, Rio de Janeiro, RJ, Brazil

**Keywords:** occupational health, nursing, intensive care units, psychosocial impact., saúde ocupacional, enfermagem, unidades de terapia intensiva, impacto psicossocial.

## Abstract

Given the evolving dynamics of the labor market, characterized by increasing work intensity and precarious conditions, analyzing the perception of psychosocial factors in the work of nursing staff in intensive care units has become an ongoing necessity. This study aimed to assess the psychosocial factors related to the work of nursing staff in intensive care units, as reported in the literature. An integrative literature review was conducted in December 2023 using the Base de Dados em Enfermagem, Virtual Health Library, Literatura Latino-Americana em Ciências da Saúde, PubMed, Index Psicologia - Periódicos, BMC Medical Ethics, Web of Science, Cumulative Index to Nursing and Allied Health Literature, and Scopus. The guiding question was: “How do psychosocial factors contribute to the illness of nursing staff in intensive care units?” A total of 21 articles published between 2000 and 2021 were included, with a predominance of Brazilian studies using qualitative methodologies. The findings revealed a phenomenon previously overlooked or not recognized as a significant issue, despite the historical presence of psychosocial factors in the nursing profession. The reviewed studies indicate psychosocial risks directly impact the health of nursing staff. There is a need for further research, particularly intervention studies, longitudinal studies, and causal relationship analyses. Additionally, implementing continuous education strategies in health care services is essential to prevent health issues caused by psychosocial factors.

## Abstract (pt)

Diante das mudanças do mundo do trabalho, com intensificação da precarização das relações de trabalho, analisar a percepção de fatores psicossociais entre profissionais de enfermagem atuantes em unidades de terapia intensiva tornou-se uma necessidade presente e constante. O objetivo do presente estudo foi avaliar, a partir de relatos da literatura, os fatores psicossociais relacionados ao trabalho das equipes de enfermagem atuantes em unidades de terapia intensiva. Foi realizada uma revisão integrativa da literatura, em dezembro de 2023, nas seguintes bases de dados: Bases de Dados em Enfermagem, Biblioteca Virtual em Saúde, Literatura Latino-Americana em Ciências da Saúde, PubMed e Index Psicologia - Periódicos, BMC Medical Ethics, Web of Science, Cumulative Index to Nursing and Allied Health Literature e Scopus. A revisão foi feita a partir da pergunta: “Como os fatores psicossociais contribuem para o adoecimento de trabalhadores de enfermagem das unidades de terapia intensiva?” Com a inclusão de 21 artigos publicados entre 2000 e 2021, com predomínio de estudos brasileiros e de metodologia qualitativa, revelou-se um fenômeno antes oculto ou pouco reconhecido como um problema relevante, apesar de os fatores psicossociais estarem historicamente presentes e acompanharem o desenvolvimento da profissão de enfermagem. Os estudos indicam que os riscos psicossociais impactam diretamente na saúde dos trabalhadores de enfermagem. No entanto, há necessidade de realização de pesquisas de intervenção, estudos longitudinais e análise de relações de causalidade, além da adoção de estratégias de educação permanente nos serviços como estratégia de prevenção de agravos à saúde pelos fatores psicossociais.

## INTRODUCTION

A broad analysis of Western societies reveals that the world of work has undergone continuous transformations, including the adoption of more flexible work structures and technological advancements. These changes impose new demands on individuals, affecting their health and quality of life.^1^ However, the full impact of these transformations remains unclear, highlighting the need for research on contemporary work and its numerous related factors to better understand the complexities of different labor contexts.

There is no denying the existence of a “24-hour society,” in which workers are exposed to psychosocial work factors influencing both health promotion and the occurrence of adverse health outcomes.^2^

The term *“*creative work scheduling*”* has been used to adjust work hours to meet the needs of the workforce. It is also employed to manage production fluctuations, worker absences, and customer demands. As a result, flexible work schedules are increasingly adopted, allowing for the expansion or reduction of working hours as needed.^3^

The European model from the 1900s, in which companies replaced traditional weekly working hours with fixed periods where employees were scheduled to work on specific days or weeks each month-or even based on annual working hours-remains a common practice today. This approach is also applied in the health care sector.^4^

By the 2010s, discussions had already highlighted how the risks of illness and the strategies to cope with resource shortages and lack of support in health care work were further exacerbating psychosocial illness, particularly in the context of mental workload.^5^

With the emergence of coronavirus disease (COVID-19) in 2020, social distancing, isolation measures, uncertainties, and the disruptions caused by the public health emergency-along with the intensified workload in high-complexity care units-further exacerbated the challenges faced by health care workers worldwide, particularly the nursing staff.^6^-^10^

Thus, analyzing the perception of psychosocial factors in the work of nursing staff in intensive care units (ICUs) has become an ongoing necessity. This professional group is among the most significantly impacted in terms of work organization, with consequences for the broader health care team. These range from minor issues, such as absenteeism, to severe outcomes like mental illness and permanent disability leave.

Moreover, addressing this issue aligns with the research priorities set by the Brazilian Ministry of Health, specifically in Axis 1 - Environment, Work, and Health, under section 1.1 - Assessment of the economic impact on the Brazilian Unified Health System (SUS) related to work-related accidents, illnesses, and health conditions.

Thus, this study aimed to assess the psychosocial factors related to the work of ICU nursing staff as reported in the literature.

## METHODS

This study is an integrative literature review (ILR) analyzing relevant studies that support decision-making and improve health care practice. This method allows for synthesizing knowledge on a specific topic while identifying gaps that require further research.^11^,^12^ The six stages of the ILR methodology were followed: formulating the review question, searching the literature in databases, data collection, critical analysis of the included studies, discussion of the results, and synthesis of knowledge.^13^

The review problem was structured using the PICO strategy, which guided the development of the review question: “How do psychosocial factors contribute to the illness of nursing staff in intensive care units?” (Chart 1).

**Chart 1:** PICO strategy, Rio de Janeiro, Brazil, 2023

Component	Definition
Population	Nursing staff
Issue	Psychosocial illness
Context	Intensive care unit work
Outcome	Contributing factors to psychosocial illness

Next, the following descriptors were defined: “Intensive Care Units,” “Occupational Health,” “Working Conditions,” “Psychosocial Support,” “Psychosocial Risks,” and “Mental Health,” along with their English equivalents, without temporal restrictions. Searches were conducted between May and September 2022, then reviewed and updated in December 2023. Boolean operators [AND] and [OR] were used to combine descriptors in pairs and later in groups of three, also without temporal restrictions. The filters “Occupational Health” and “Healthcare Personnel” were applied.

Searches were conducted in the following databases: Base de Dados em Enfermagem (BDEnf), Virtual Health Library (BVS), Literatura Latino-Americana em Ciências da Saúde (LILACS), PubMed, Index Psicologia - Periódicos, BMC Medical Ethics, Web of Science, Cumulative Index to Nursing and Allied Health Literature (CINAHL), and Scopus. Such sources were selected due to their global coverage of scientific journals, following the search syntax outlined in Chart 2.

**Chart 2:** Summary of descriptors and Boolean operators used in literature searches, Rio de Janeiro, Brazil, 2023

Databases	Search syntax used
Literatura Latino-Americana em Ciências da Saúde (LILACS)	Unidade de terapia intensiva, [AND], saúde do trabalhador n = 11 suporte psicossocial, [AND], saúde mental n = 16 suporte psicossocial, [AND], saúde mental, [AND], unidade de terapia intensiva n = 1 condições de trabalho,[OR], saúde do trabalhador,[AND] unidade de terapia intensiva adulta n=12 Riscos Psicossociais,[AND], Unidade de terapia intensiva n = 10
Bases de Dados em Enfermagem (BDEnf)	Unidade de terapia intensiva, [AND], saúde do trabalhador n = 2 suporte psicossocial, [AND], saúde mental n = 6 suporte psicossocial, [AND], saúde mental, [AND], unidade de terapia intensiva n = 1 condições de trabalho,[OR], saúde do trabalhador, [AND], unidade de terapia intensiva adulta n = 13 suporte psicossocial, [AND], Trabalhadores de saúde n = 3 Riscos Psicossociais, [AND] Unidade de terapia intensiva n = 7
Index Psicologia - Periódicos	Unidade de terapia intensiva, [AND], saúde do trabalhador n = 1 suporte psicossocial, [AND], saúde mental n = 2 condições de trabalho, [OR], saúde do trabalhador, [AND] unidade de terapia intensiva adulta n = 0 Riscos Psicossociais, [AND], Unidade de terapia intensiva n = 2
PubMed	Unidade de terapia intensiva, [AND], saúde do trabalhador n = 0 suporte psicossocial, [AND], saúde mental n = 0 suporte psicossocial, [AND], saúde mental, [AND], unidade de terapia intensiva n = 0 condições de trabalho, [OR], saúde do trabalhador, [AND] unidade de terapia intensiva adulta n = 0 Riscos Psicossociais, [AND], Unidade de terapia intensiva n = 0
BMC Medical Ethics	Unidade de terapia intensiva, [AND], Saúde mental n = 1
Scopus	( title-abs-key ( intensiveandcareandunit )andtitle-abs-key ( occupationalandheath )andtitle-abs-key ( psychosocialandfactors ) n = 0
Cumulative Index to Nursing and Allied Health Literature (CINAHL)	intensive care unit (AND) occupational health (AND) psychosocial factors n=0
Web of Science	occupational health,( AND) psychosocial factor ( AND) intensive care unit n = 19

**Figure 1:**
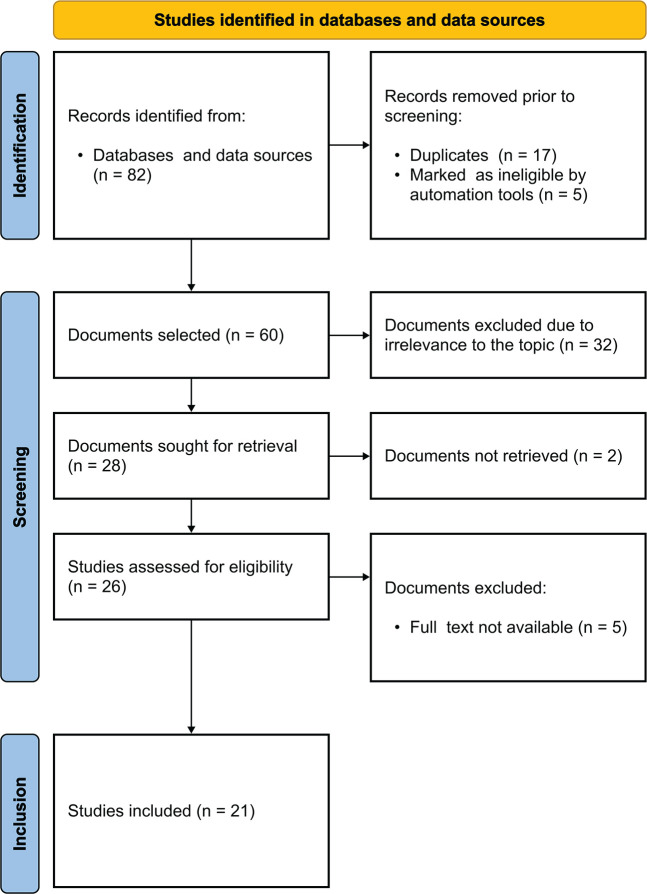
Flowchart of article selection, Rio de Janeiro, Brazil, 2023. Source: Adapted from Page et al.^13^

Article selection followed the guidelines outlined in the Preferred Reporting Items for Systematic Reviews and Meta-Analyses (PRISMA) framework^13^ (Figure 1).

Articles included in this review comprised research studies, reflective texts, and literature reviews, given their potential to generate new scientific evidence. The selected studies focused on occupational health and the nursing staff in the context of psychosocial illness, with no language restrictions. Articles that did not address the primary study objective or were not published in peer-reviewed journals were excluded.

## RESULTS AND DISCUSSION

Following the search and selection process, 21 studies were included. A summary of their content is presented in the synoptic table (Chart 3).

**Chart 3:** Summary of included studies after database search, Rio de Janeiro, Brazil, 2023

Year of publication Country	Study design	Summary of results
2000 Brazil^14^	Quali-quantitative	Psychosocial factors were analyzed based on the most valued quality-of-life aspects, with a positive evaluation, though differences were observed between two nursing teams.
2002 Brazil^15^	Qualitative	From the perspective of social representations, various psychological defense mechanisms are used individually to cope with patient pain, suffering, and death.
2009 Brazil^16^	Qualitative	Workloads within the institution cause physical and mental strain, leading to alienation regarding quality of work life. This results in emotional conflicts, stress, and physical pain. Workers feel discouraged or incapable of providing quality care, leading to psychological distress.
2009 Brazil^17^	Quantitative	Nursing is considered one of the most stressful hospital professions due to the complexity of tasks requiring high attention and dedication. An individualized approach to prevent burnout is suggested, considering each professional’s perception of stressors in their work environment.
2010 Brazil^18^	Literature review	Predisposing factors for stress include work overload, role conflicts, devaluation, and working conditions. Symptoms include tachycardia, loss of appetite, chills, anxiety, and joint pain.
2011 Portugal^19^	Quantitative	Men, a minority in the study group, perceived higher levels of psychosocial risks. Individuals over 50 years old, those who were married or in a partnership, reported a greater perception of health risks. Working in hospitals was associated with a higher risk of job dissatisfaction related to social and family support. Strong social support was linked to a lower perception of risks, while self-concept played a role in reducing risks related to personal values and both physical and psychological health.
2011 Brazil^20^	Quantitative	Critical levels of factors contributing to feelings of pleasure, professional fulfillment, and freedom of expression were identified. At the same time, factors causing distress, professional burnout, and lack of recognition were also found at critical levels among nurses and nursing technicians.
2012 Brazil^21^	Qualitative	Key findings, analyzed through content analysis, include low work recognition, intensified workload leading to overload, ethical dilemmas between personal values and professional demands, institutional rigidity, and distress related to patient deaths.
2013 Brazil^22^	Literature review	Few studies were found on stressors and preventive measures for stress, highlighting a gap in essential care for promoting worker health.
2013 Brazil^23^	Qualitative	Nurse residents in specialized hospital units are exposed to numerous psychosocial risk factors, including physical and mental workload, intensified work pace, role ambiguity, interpersonal conflicts, low autonomy, limited control, and precarious working conditions. The health impacts on residents were identified through complaints of fatigue, stress, and exhaustion.
2013 Brazil^24^	Quantitative	Key issues identified included low work recognition and support, work overload, night shifts (causing sleep disturbances), difficulties in relationships with supervisors, ethical dilemmas between personal values and professional responsibilities, institutional rigidity, and challenges in coping with patient deaths. Coping strategies observed among workers included denial, trivialization of suffering, rationalization, and avoidance.
2014 Brazil^25^	Quantitative	Low intellectual discernment, weak social support, and experiencing either highly demanding or passive work were identified as the main risk factors for impairments in the physical domain of quality of life.
2014 Brazil^26^	Qualitative	Workloads within the institution were found to cause physical and mental exhaustion, leading to emotional conflicts and health repercussions, such as stress and bodily pain.
2014 Brazil^27^	Quantitative	Regarding pleasurable factors, freedom of expression received a satisfactory evaluation, while professional fulfillment was assessed critically. For distress factors, professional burnout was critically evaluated, whereas lack of recognition was considered satisfactory.
2015 Chile^5^	Quantitative	ICU nurses perceive psychosocial factors and mental workload across multiple dimensions of their work.
2015 Brazil^28^	Quantitative	The prevalence of burnout syndrome was 55.3% (n = 72), while 27.7% of cases were suspected common mental disorders. Among these, 80.6% were associated with burnout (p < 0.0001). Income and thinking about work during time off were identified as protective factors against burnout in moderate-stress active work (OR = 0.26; 95% CI: 0.09-0.69) and passive work (OR = 0.22; 95% CI: 0.07-0.63). The findings confirmed that psychosocial factors were involved in the development of burnout in the studied group.
2018 Brazil^29^	Quantitative	Preventive measures are essential to control mental health disorders and prevent unnecessary harm to nursing professionals’ health, quality of life, institutions, and even the social security system. While stress can act as a stimulus for new challenges, the organization and nature of nursing work contribute to the insidious development of burnout.
2018 England^30^	Reflection	A 100% positive response was reported for the reflective debriefing intervention, which emphasized the importance of interprofessional collaboration in successfully addressing moral distress among health care workers. This approach may help protect workers from burnout, emotional detachment, and even leaving the profession.
2020 Brazil^31^	Literature review	The importance of the nursing staff’s work must be recognized across all health care settings, especially in emergency situations. Therefore, in addition to adequate working conditions, psychosocial support is essential for preserving these professionals’ mental health, benefiting both workers’ well-being and the quality of patient care.
2020 Brazil^5^	Quantitative	Health care workers working in hospital settings are exposed to various occupational stressors that directly impact their well-being, including long work hours and constant exposure to pain, suffering, and death. There is a critical need to promote workers’ mental health to ensure optimal professional performance and deliver high-quality patient care.
2021 The Netherlands^34^	Quantitative	The levels and causes of moral distress vary among ICU workers and differ from those observed in the historical control group. Targeted interventions addressing moral distress during crises are desirable to improve mental health, enhance ICU staff retention, and maintain the quality of patient care.

ICU = intensive care unit; OR = odds ratio.

The findings, published between 2000 and 2021, reveal a phenomenon previously overlooked or not recognized as a significant issue, despite the historical presence of psychosocial factors in the nursing profession.

Most studies were conducted in Brazil (n = 18),^5^,^14^-^18^,^20^-^29^,^31^ with additional research on nursing from Portugal (n = 1),^19^ Chile (n = 1),^5^ England (n = 1),^30^ and the Netherlands (n = 1).^32^ This distribution is notable, particularly considering the study period could have included health impacts related to the COVID-19 pandemic.

However, the Brazilian health care landscape has shown, for over a decade, an increase in precarious employment conditions in health care and nursing, along with work intensification and overload.^33^-^35^

During the same period, it was already acknowledged workplace accidents and occupational diseases could be exacerbated by social, political, economic, and cultural factors related to nursing work,^36^,^37^ yet such aspects appear to have received limited attention in the in-depth reflection on the relationship investigated in the study results.

Qualitative studies were predominant, followed by quantitative studies and literature reviews. This trend may be related to the difficulty of capturing the phenomenon through a positivist perspective since it involves a complex framework encompassing interactions between workers, the general environment, and the workplace.^38^

From the data analyzed, the main challenges faced by nursing professionals in their intensive care roles were identified, with a particular emphasis on social and psychological factors.

ICUs are hospital units designed for critically ill and unstable patients, often representing a high-stress environment. These units require specialized technical procedures, advanced equipment and materials, and constant attention from health care workers.^39^

Nursing professionals in ICUs are exposed to physical, chemical, biological, social, and psychological risks, classified as environmental and occupational hazards. Such factors create a work environment that can disrupt physical, mental, and social well-being, regardless of whether they lead to accidents or illnesses.^5^

Workplace stress is directly related to the lack of structured operational workflows that support patient care. This deficiency leads to work overload for nursing staff, strain in professional relationships, and a significant increase in errors and lapses in care due to the high demand and workload.^5^

In ICUs, occupational risks may be further intensified due to the fast-paced, complex, and specialized nature of the unit. Consequently, nursing staff health in ICUs must be continuously monitored.^29^ Fatigue serves as a warning signal, prompting the body to recognize its limits and require rest. When this need for recovery is ignored, professionals become physically and mentally exhausted.^28^

The intensive care setting demands highly trained and specialized professionals who must work under significant physical and psychological strain - a combination that can lead to work-related distress and illness.^25^

Stress is a significant factor that negatively impacts professional performance, leading to perceptual errors and difficulty concentrating on tasks.^30^ Stress is closely linked to working conditions, including complex tasks requiring immediate decision-making, long working hours affecting rest, a shortage of skilled nursing professionals, and the resulting work overload. Another major source of stress is physical and/or verbal aggression from patients, relatives, and visitors in ICUs.^27^

Nursing is a profession that involves constant exposure to suffering and emotional strain, requiring special attention, compassion, and empathy. Feelings of powerlessness and hopelessness can be incompatible with professional performance, contributing to guilt and anxiety.^21^

Additionally, the ICU environment is often described as unhealthy, influencing the attitudes and habits of nursing professionals. This highlights the need for institutional investment in educational approaches focused on worker health.^33^

However, educational initiatives targeting worker health remain scarce, despite their crucial role in reducing occupational risks. This underscores the need for improvements in working conditions, professional training for critical care settings, and the development of specific actions, strategies, and protective measures to help mitigate occupational hazards for nursing staff.^41^

Hard technology (technological apparatus) is also considered a psychosocial risk factor with negative health impacts on workers. It has been identified as a source of occupational distress, contributing to professional complaints such as anxiety, stress, fatigue, and exhaustion due to work-related conditions.^26^

There is a growing use of validated scales to assess mental, emotional, and psychosocial health issues among ICU workers. These tools help identify work-related distress, satisfaction, and stress, potentially guiding changes in nursing work environments. However, few studies have presented assessments or interventions specifically targeting ICU nursing staff.^35^ Moreover, only a few studies have reported the use of these measurement tools, including the Flanagan Quality of Life Scale (QOLS),^14^ the Job Stress Scale (JSS),^28^ the Measure of Moral Distress for Health Care Professionals (MMD-HP), and the Ethical Decision-Making Climate Questionnaire (EDMCQ).^32^ However, these instruments do not directly assess the core phenomenon under study.

Finally, there is a predominance of cross-sectional and descriptive studies, highlighting a gap in longitudinal research on the long-term impact of psychosocial factors on workers’ health. Additionally, intervention studies are scarce, with only one international study including such an approach.^29^ Moreover, no studies were found that established potential causal relationships between psychosocial factors and their effects.

## CONCLUSIONS

The studies indicate that psychosocial risks directly impact the health of nursing staff, with both physical and mental repercussions standing out among the publications analyzed.

Regarding the effects of psychosocial factors on the health of ICU nursing staff, there is a clear need for intervention studies, longitudinal research, and causal relationship analyses. Additionally, evaluating the impact of continuous education strategies in health care services is crucial. Such strategies, mediated by nurses in intensive care units, have the potential to raise awareness and empower workers in this sector, promoting health protection and reducing the psychosocial burden associated with patient care.

The use of validated assessment tools is essential for targeted and evidence-based evaluations, but results from such applications have yet to be widely published in the literature.

There is an urgent need to bridge the significant gap between nursing practice and the prevention of health problems in relation to psychosocial factors. This can be achieved through ongoing professional education and engagement by both nurses and their health managers, whether through literature review or the development of individual skills aimed at mitigating the risks associated with psychosocial stressors in the workplace.
